# Association between serum ferritin and the severity of drug eruptions

**DOI:** 10.1002/jcla.23043

**Published:** 2019-09-25

**Authors:** Yunlei Pan, Shunli Tang, Siting Zheng, Sheng Li, Dingxian Zhu, Hong Fang, Jianjun Qiao

**Affiliations:** ^1^ Department of Dermatology The First Affiliated Hospital Zhejiang University Hangzhou China

**Keywords:** drug eruptions, ferritin, severe cutaneous adverse drug reaction

## Abstract

**Background:**

Early recognition and treatment for severe drug eruption are important in improving clinical outcomes. A few studies have reported laboratory parameters to evaluate the severity of drug eruptions. This study aimed to determine the association between serum ferritin and the severity of drug eruptions.

**Methods:**

We retrospectively reviewed patients diagnosed with drug eruptions in our hospital from 2013 to 2018.

**Results:**

We identified 85 patients (mean age 53.4 years), 20 in the severe cutaneous adverse drug reactions (SCADRs) group and 65 in the non‐SCADRs group. Serum ferritin level was higher in the SCADRs group compared with that in the CADRs group (*P*<.001). Serum ferritin was positively associated with peripheral white blood cell count, aspartate aminotransferase level, alanine aminotransferase level, blood glucose level, blood creatinine level, and body temperature. Receiver operating characteristic (ROC) analysis revealed a good diagnostic value of ferritin (area under the curve [AUC]:0.87, 95% confidence interval [CI]:0.78‐0.96) with a sensitivity of 80% and a specificity of 87.7% at a cutoff value of 416.15 ng/mL.

**Conclusions:**

Serum ferritin is significantly associated with the severity of CADRs and hence might be potentially used to evaluate the severity of CADRs.

## 1 INTRODUCTION

Drugs have adverse effects including cutaneous adverse drug reactions (CADRs), which are frequent in modern drug therapy and affect 2% to 3% of all hospitalized patients.^1^, ^2^ There are mild and moderate forms of CADRs, such as lichenoid eruptions, maculopapular exanthems (MPE), urticaria, and fixed drug eruption (FDE), while severe forms of CADRs include acute generalized exanthematous pustulosis (AGEP), Stevens‐Johnson syndrome (SJS), toxic epidermal necrolysis (TEN), and drug reactions with eosinophilia and systemic symptoms (DRESS)/hypersensitivity syndrome (HSS), which are potentially fatal and account for a high mortality from 10% to 40%.^3^, ^4^ Recognizing the severity of CADRs and initiating appropriate treatment at an early stage are important in improving clinical outcomes. However, the previous studies showed that the early diagnosis of CADRs was significantly difficult.^5^, ^6^

Ferritin is an iron storage protein influenced by the intracellular iron concentrations. It is also an acute‐phase protein, which is increased in the setting of inflammation, even exceeding 10 000 ng/mL in septic shock and infectious diseases with macrophage activation.^7^, ^8^, ^9^ Increased ferritin level is also observed in alcoholism, cytolysis (including hepatic and muscle cytolysis), metabolic syndrome, juvenile idiopathic arthritis, and adult‐onset Still's disease.^10^, ^11^, ^12^ One study revealed that ferritin could be a useful early prognostic factor in DRESS.^13^ Thus, we determine the association between serum ferritin and the severity of drug eruptions.

## 2 MATERIALS AND METHODS

We conducted a retrospective study of in 166 inpatients who were diagnosed with drug eruptions in the Department of Dermatology from July 31, 2013, to July 31, 2018. Patients with iron deficiency, iron deficiency anemia, and iron overload were excluded. Other exclusion criteria were as follows: conditions that could potentially affect the body's iron storage or ferritin including any acute or chronic inflammatory conditions, hemorrhagic disorders, pregnancy, hemoglobinopathies, cancer, diabetes, and liver disease. Patients who met the inclusion criteria were divided into the following two groups: the SCADRs group and the non‐SCADRs group. The SCADRs group included patients who were diagnosed with SJS/TEN, DRESS/HSS, and AGEP, while the non‐SCADRs group was consisted of patients who were diagnosed with MPE, FDE, lichenoid eruptions, and urticaria. Diagnoses were established according to the respective diagnostic criteria.^14^ This study protocol was approved by the local ethics committee of our hospital.

We searched data from the medical records, including clinical symptoms, lesion distribution and morphology, laboratory findings, comorbid diseases, and medications. Some clinical variables that were retrieved and recorded were as follows: pulse rate (PR), body temperature (BT), peripheral white blood cell (WBC) count, eosinophil count, serum ferritin level, serum albumin (ALB) level, aspartate aminotransferase (AST) level, alanine aminotransferase (ALT) level, blood glucose (GLU) level, blood creatinine (CR) level, and blood urea nitrogen (BUN) level. Chemiluminescence microparticle immuno assay was used to assess serum ferritin level. The modified systemic inflammatory response syndrome (mSIRS) was used to evaluate the inflammatory status. It included WBC count, PR, BT, and respiratory rate, with the lowest score of 0 and highest score of 3 in each parameter.

### 2.1 Statistical analysis

Statistics analysis was performed using SPSS ver. 19 statistical software. Intergroup comparison of the difference was performed using Mann‐Whitney *U* test. The association between ferritin and other variables was performed using Pearson's correlation analysis. The association between ferritin level and mSIRS score was executed by Spearman's rank correlation test. The diagnostic value of ferritin level was evaluated using the receiver operating characteristic (ROC) curve. The test sensitivity, specificity, and cutoff value were estimated according to the Youden criteria. *P* value <.05 was considered to be statistically significant.

## 3 RESULTS

**Figure 1:**
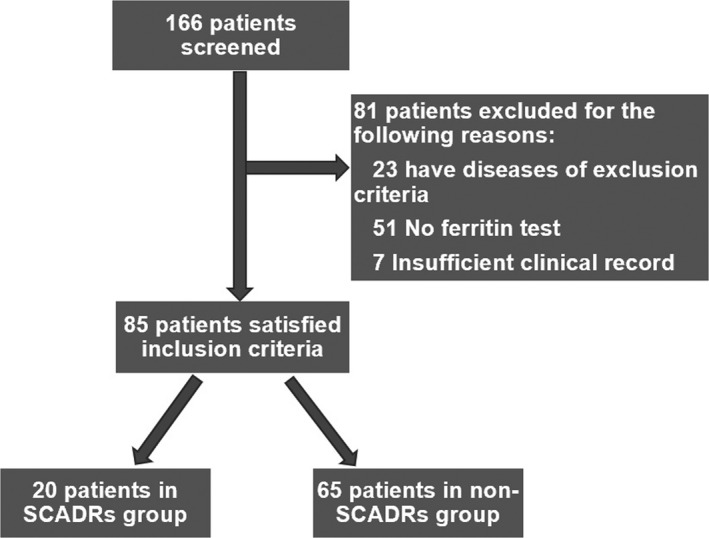
Patients selection and classification. SCADRs, severe cutaneous adverse drug reactions

**Figure 2:**
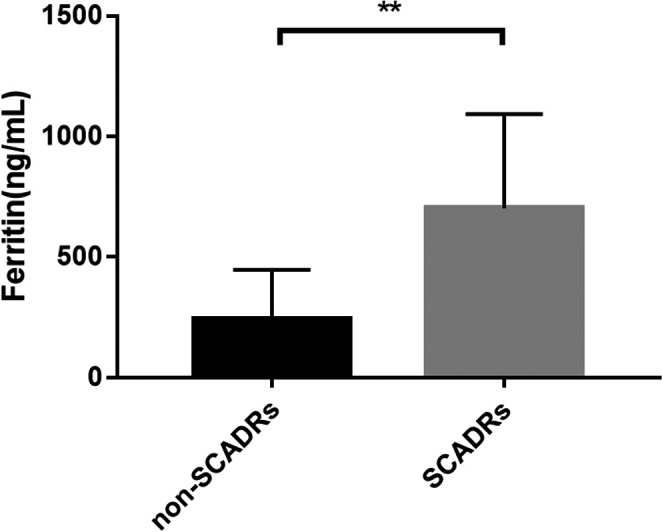
Serum ferritin in severe cutaneous adverse drug reactions (SCADRs) group (n = 20) and non‐SCADRs group (n = 65). Serum ferritin level was significantly higher in SCADRs group compared with non‐SCADRs group (*P*<.001)

**Figure 3:**
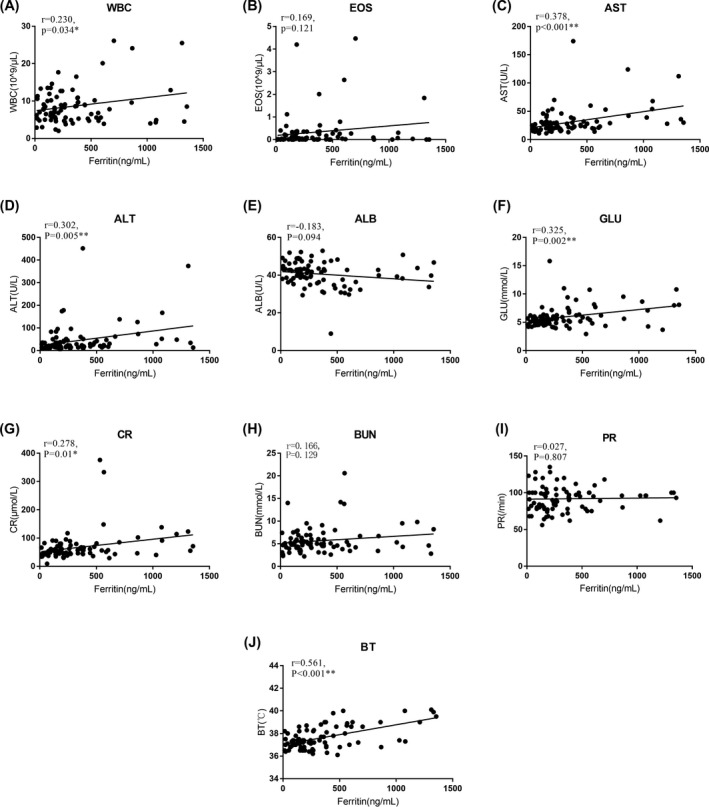
Correlation between serum ferritin and clinical variables. Correlation between serum ferritin and peripheral white blood cell count (WBC), eosinophil count (EOS), aspartate aminotransferase (AST), alanine aminotransferase (ALT), serum albumin (ALB), blood glucose (GLU), blood creatinine (CR), blood urea nitrogen (BUN), pulse rate (PR) (*P* < .05* and *P* < .01**)

Eighty‐five patients aged was 53.4 ± 17.39 years (mean ± SD), were included in our study, with 81 patients excluded. Twenty patients were part of the SCADRs group, and 65 patients were part of the non‐SCADRs group (Figure 1). There was no significant difference in age and sex between the two groups. All laboratory tests including ferritin level were performed at initial presentation to our hospital. Ferritin levels were 703.19 ± 390.36 ng/mL in the SCADRs group and 244.90 ± 201.41 ng/mL in the non‐SCADRs group. Statistically significant differences were observed between the two groups (*P*<.001; Figure 2). Comparison of clinical and laboratory features of patients between the two groups is shown in Table 1. In our study, the WBC, AST, ALT, GLU, CR, and BT were positively associated with ferritin level at the time of diagnosis. On the contrary, ALB, EOS, BUN levels, and PR were not significantly associated with ferritin level at the time of diagnosis (Figure 3). Moreover, serum ferritin level was positively associated with mSIRS score (*r* = .589, *P *< .001).

**Table 1:** Clinical variables of the selected patients

Parameters	SCADRs group (n = 20)	non‐SCADRs group (n = 65)	*P* Value
Female/Male (NO.)	9/11	38/27	.112
Age (y), (mean ± SD)	57.35 ± 16.65	52.2 ± 17.56	.170
BT (°C), mean ± SD	38.73 ± 1.07	37.30 ± 0.71	<.001
PR (/min), mean ± SD	98.15 ± 18.98	89.83 ± 16.32	.109
WBC count (10^9^/μL), mean ± SD	12.04 ± 7.74	7.62 ± 3.03	.094
eosinophil count (10^9^/μL), mean ± SD	0.69 ± 1.23	0.22 ± 0.59	.700
Ferritin (ng/mL), mean ± SD	703.19 ± 390.36	244.90 ± 201.41	<.001
ALB (U/L), mean ± SD	35.10 ± 7.96	42.24 ± 5.39	<.001
AST (U/L), mean ± SD	34.95 ± 21.68	29.56 ± 25.24	.040
ALT (U/L), mean ± SD	61.45 ± 84.89	40.18 ± 62.24	.054
GLU (mmol/L), mean ± SD	6.61 ± 2.09	5.78 ± 1.89	.064
CR (μmol/L), mean ± SD	100.40 ± 91.84	57.14 ± 21.77	.012
BUN (mmol/L), mean ± SD	7.24 ± 4.11	5.17 ± 2.20	.010

Abbreviations: ALB, serum albumin; ALT, alanine aminotransferase; AST, aspartate aminotransferase; BT, body temperature; BUN, blood urea nitrogen; CR, blood creatinine; GLU, blood glucose; PR, pulse rate; SCADRs, severe cutaneous adverse drug reactions; WBC, white blood cell.

**Figure 4:**
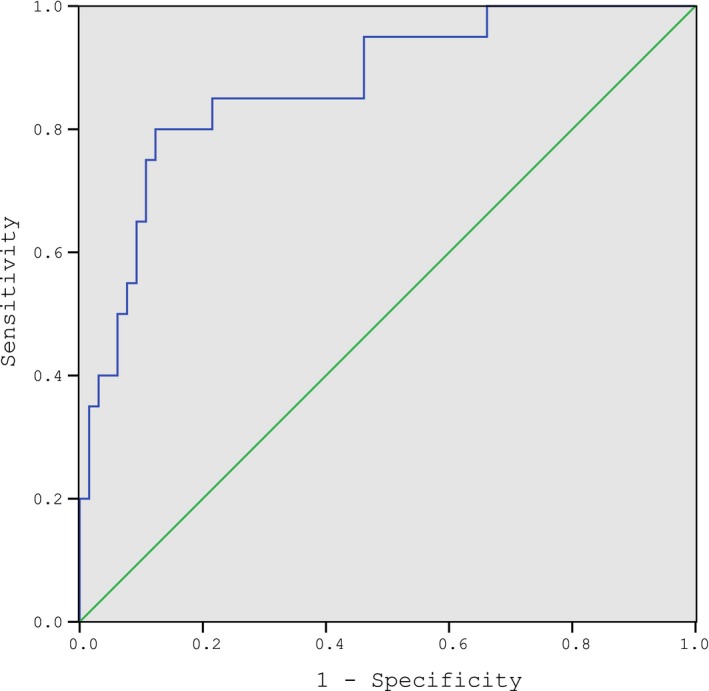
A receiver‐operator characteristic curve analysis of ferritin in drug eruptions. Area under the ROC curve values of 0.87 (95% CI:0.78‐0.96), Cutoff value 416.15 ng/mL, sensitivity 80% specificity 87.7%

Based on our observation, higher level of serum ferritin was associated with a more severe outcome. We presumed that ferritin level could be potentially used to evaluate the severity of the disease condition. Thus, a ROC analysis was performed to assess serum ferritin level. According to the analysis, the AUC was 0.87 (95% CI:0.78‐0.96). Serum ferritin level had a sensitivity of 80% and a specificity of 87.7% with a cutoff value of 416.15 ng/mL according to the Youden criteria (Figure 4).

## 4 DISCUSSION

With drug consumption increasing worldwide, the CADRs are becoming increasingly common. Although only a small proportion of CADRs are severe and life‐threatening, patients with CADRs are severely affected, with significant economic impact in their lives. Early identification of CADRs and initiation of appropriate treatment are helpful in achieving better clinical outcomes and in reducing inpatient fee. Our study revealed that serum ferritin level has a predictive value for the severity of drug eruptions in the early stage. It was significantly higher in the SCADRs group compared with the non‐SCADRs group, a result that is consistent with some case reports and studies showing the association between hyperferritinemia and drug eruptions in SJS and DRESS.^15^, ^16^, ^17^ The association between ferritin and some variables, which may reflect the severity of drug eruptions and physical condition of patients, was observed. A ROC analysis revealed that serum ferritin had high sensitivity and specificity in predicting SCADRs.

The mean age of patients was 53.4 years, which is considered an advanced age and could be due to the fact that elderly people are more susceptible to various diseases and have higher chances of taking medicine; thus, drug eruption is more common in this age group than other age groups. It showed that BT of patients was higher in the SCADRs group than in the non‐SCADRs group, and ALB level was lower in the SCADRs group compared with the non‐SCADRs group. Patients with SCADRs had inflammation, hence increasing their BT. In this process, the SCADRs group consumed higher ALB level than the non‐SCADRs group. Ferritin level was significantly associated with mSIRS score, which is one of the indicators of systemic inflammation. The SIRS score was used to assess the mortality rate of patients in the intensive care units. Our findings were also supported by several reports showing that ferritin was associated with inflammation.

Although the findings of our study were encouraging, the mechanism underlying the increase of serum ferritin level in drug eruptions was unclear. Drug eruptions are usually considered as immunologically mediated reactions, and the immunopathogenesis involves several inflammatory and cytotoxic mediators.^18^, ^19^ On the contrary, there are studies that showed that serum ferritin level increased in the inflammation.^20^, ^21^ Hence, it probably was caused by the different inflammatory mediators such as cytokines, which were associated with the drug eruptions, specifically the severe forms of drug eruptions.^18^, ^22^ The limitation of our study was that this is a retrospective analysis with limited number of patients. Thus, a prospective study including more patients to identify the sequence of ferritin level increase and SCADRs occur is significantly required. Another limitation was that the number of C‐reactive protein test of patients was insufficient in performing the analysis.

In conclusion, our study showed that serum ferritin levels were positively associated with the severity of CADRs at the time of diagnosis. Ferritin detection might be a screening tool for the severity of CADRs.

## CONFLICT OF INTEREST

The authors declared no conflicts of interest.
